# Evolving patterns of bladder cancer mortality in US adults: a two-decade analysis reveals persistent disparities despite overall progress (1999-2020)

**DOI:** 10.3389/fonc.2026.1713506

**Published:** 2026-03-02

**Authors:** Lingfeng Sun, Chengyi Liu, Lin Yuan

**Affiliations:** 1 The First Clinical Medical College, Nanjing University of Chinese Medicine, Nanjing, China; 2 Department of Urology, Affiliated Hospital of Nanjing University of Chinese Medicine, Jiangsu Province Hospital of Chinese Medicine, Nanjing, China; 3 Department of Urology Department of Urology, LU’AN Hospital of Anhui Medical University, Lu’an, Anhui, China

**Keywords:** bladder cancer, CDC WONDER, epidemiology, mortality, trends

## Abstract

**Objective:**

Bladder cancer represents one of the most common malignancies in the United States, with mortality trends reflecting disease burden and clinical management effectiveness. This study aimed to comprehensively analyze long-term trends and demographic disparities in bladder cancer mortality among US adults from 1999 to 2020 using national data.

**Methods:**

Mortality data with bladder cancer (ICD-10 code: C67) as the underlying cause of death were extracted from the CDC WONDER database for 1999-2020.AAMR was calculated. Joinpoint regression models were employed to calculate APC and AAPC to identify temporal trends and inflection points in mortality rates. Stratified analyses were conducted by sex, age group, race/ethnicity, urbanization level, and geographic region.

**Results:**

From 1999 to 2020, a total of 319,229 adult deaths in the United States had bladder cancer as the underlying cause of death. Overall age-adjusted mortality rates declined from 6.737 per 100,000 in 1999 to 6.159 per 100,000 in 2020 (AAPC: -0.410, 95% CI: -0.495 to -0.310, P < 0.001). Joinpoint regression analysis revealed a significant decline trend after 2016 (APC changed from -0.347 to -2.118). Stratified analysis showed that male mortality rates were significantly higher than female rates. White populations had the highest recorded mortality rate (7.003 per 100,000), though this likely reflects diagnostic coding biases rather than true biological differences. Regional disparities existed, with the Northeast showing the highest mortality rates. Non-metropolitan residents had higher mortality rates (6.887 per 100,000) compared to metropolitan residents (6.64 per 100,000).

**Conclusion:**

Over the past two decades, bladder cancer mortality among US adults has demonstrated an overall declining trend, reflecting advances in diagnostic and therapeutic technologies. However, significant disparities by sex, race, and geographic region persist, indicating prominent health inequality issues. Future efforts should focus on strengthening early screening, smoking cessation interventions, and standardized treatment for high-risk populations to further reduce disease burden.

## 1 Introduction

Globally, bladder cancer ranks as the tenth most common malignancy by incidence and thirteenth by mortality, accounting for approximately 573,000 new cases and 212,000 deaths annually worldwide (1). Within the United States, it represents the fourth most common cancer among men and the eleventh among women, resulting in approximately 83,730 new cases and 17,200 deaths each year (2). The burden of this disease extends well beyond its high incidence rates, encompassing significant treatment complexity, elevated recurrence rates of 50-70%, and the substantial economic impact of lifelong surveillance. Estimates indicate that lifetime treatment costs for bladder cancer patients in developed countries range from $96,000 to $187,000 per patient notably exceeding the average expenditure for all cancer types (3). Consequently, from a public health perspective, systematic analysis of long-term bladder cancer mortality trends and their distribution across diverse population subgroups provides invaluable insights for evaluating prevention and control policies, guiding efficient resource allocation, and developing optimized intervention strategies.

Despite numerous previous investigations providing partial insights into bladder cancer epidemiology in the United States, there remains a notable scarcity of comprehensive, long-term, national-level analyses examining mortality trends since the turn of the 21st century, especially for the most recent decade (4). Existing research on bladder cancer mortality tends to be limited in both temporal and geographic scope, predominantly featuring short-term or regionally focused studies. Such limitations prevent these works from adequately capturing the dynamic evolution of the US bladder cancer mortality landscape a landscape significantly transformed by evolving tumor classification standards, the advent of molecular targeted therapies, and the revolutionary introduction of next-generation treatments such as immune checkpoint inhibitors (5, 6). Moreover, in this contemporary era characterized by unprecedented focus on health equity, conducting in-depth analyses of gender, racial, and geographic disparities in bladder cancer mortality has become imperative, as such investigations can illuminate underlying healthcare inequalities and inform the development of precisely targeted intervention strategies (7).

The primary risk factor for bladder cancer smoking has undergone significant changes in prevalence trends in the United States over the past two decades, with national adult smoking rates declining from 23.5% in 1999 to 12.5% in 2020 (8). Given that approximately 50-65% of bladder cancer cases are attributable to smoking, and the risk reduction following smoking cessation requires 10-20 years to manifest significantly, analyzing mortality trends during this period can provide critical information for evaluating the long-term impact of tobacco control policies on bladder cancer disease burden (9). Similarly, occupational exposures (such as in dye, rubber, aluminum, and paint industries) account for 10-20% of bladder cancers, and with the transformation of US manufacturing structure and improvements in occupational safety standards, their impact on bladder cancer mortality also requires evaluation through long-term trend analysis (10).Recent research has further revealed the influence of several emerging risk factors on bladder cancer pathogenesis. Dietary patterns significantly influence bladder cancer risk, with studies indicating that adherence to a Mediterranean diet may have protective effects, while consumption of processed meats may increase risk (11). Regarding environmental exposure factors, disinfection byproducts in chlorinated drinking water and certain air pollutants have been found to promote bladder cancer development through oxidative stress mechanisms and DNA damage pathways (12). Additionally, the gut microbiome has gained widespread attention as a potential mediator in bladder cancer development and progression, with research showing that bladder cancer patients exhibit distinctive microbial signatures that may influence not only disease susceptibility but also treatment response (11, 12). The identification of these emerging risk factors provides new perspectives for bladder cancer prevention and individualized risk assessment.

From a clinical practice perspective, the field of bladder cancer has witnessed remarkable advancements over the past two decades. In diagnostics, traditional cystoscopy has been complemented by the widespread implementation of innovative technologies including narrow band imaging (NBI), fluorescence cystoscopy (PDD), and urinary biomarker detection potentially enhancing early diagnosis rates (13). Treatment protocols have similarly evolved: intravesical therapy regimens for non-muscle-invasive bladder cancer (NMIBC) have undergone substantial standardization, perioperative chemotherapy for muscle-invasive bladder cancer (MIBC) has gained increasing prominence, and therapeutic approaches for advanced and metastatic disease have transformed dramatically, progressing from single-agent platinum-based chemotherapy to diverse treatment modalities encompassing immune checkpoint inhibitors, fibroblast growth factor receptor (FGFR) inhibitors, and antibody-drug conjugates (14, 15). Whether these significant clinical innovations have successfully translated into population-level survival benefits, however, necessitates verification through comprehensive mortality trend analyses derived from large-scale, nationally representative data.

Of particular significance are the pronounced gender disparities in bladder cancer epidemiology, characterized by male incidence rates that exceed female rates by approximately 3-4 fold a disparity primarily attributed to differences in smoking patterns and occupational exposure profiles (16). Nonetheless, emerging research indicates that female bladder cancer patients frequently present with more advanced disease and experience poorer prognoses. These disparities potentially reflect gender bias within primary care settings (exemplified by the misattribution of female hematuria to urinary tract infections) as well as underlying biological differences (17). Parallel disparities exist among racial groups, with African American bladder cancer patients experiencing significantly lower survival rates compared to their white counterparts a disparity that persists even after adjustments for socioeconomic status and treatment access variables, suggesting the involvement of more nuanced biological mechanisms and systemic healthcare factors (18).

The highly decentralized structure of the American healthcare system engenders considerable regional variations in bladder cancer management practices. Research demonstrates that surgical outcomes at high-volume hospitals and by experienced high-volume surgeons substantially surpass those at low-volume institutions; however, these specialized centers are inequitably distributed throughout the nation (19). Similarly, regional variations in the adoption of complex treatment approaches such as radical cystectomy combined with neoadjuvant chemotherapy or precision medicine-guided targeted therapy selection may significantly influence mortality patterns (20). Therefore, analyzing geographic variations in bladder cancer mortality trends and disparities yields valuable insights for optimizing healthcare resource allocation and standardizing clinical practices nationwide.

Consequently, a systematic analysis of bladder cancer mortality trends among US adults from 1999-2020, with particular attention to variations across diverse population subgroups, offers substantial clinical and public health value. Such analysis enables comprehensive evaluation of disease prevention and control policy effectiveness, identification of entrenched health inequalities, refinement of clinical practice guidelines, and optimization of resource allocation strategies. By leveraging the nationally representative CDC WONDER database, this study addresses a critical knowledge gap in current literature regarding long-term trends in US bladder cancer mortality, thereby establishing robust, evidence-based foundations for future prevention and control strategy development.

## 2 Methods

### 2.1 Data source

This study drew upon data from the WONDER online database (https://wonder.cdc.gov/), a comprehensive resource meticulously maintained by the Centers for Disease Control and Prevention. Widely recognized as an authoritative source for mortality and population statistics in the United States, this database encompasses exhaustive population mortality data extracted from death certificates across all fifty states and the District of Columbia. For our analytical purposes, we specifically employed the “Underlying Cause of Death” module to extract relevant mortality information (21). As the CDC WONDER database exclusively contains anonymized public data that is freely accessible for research purposes, institutional review board approval was deemed unnecessary for the conduct of this investigation.

### 2.2 Definition of study population and variables

Our investigation encompassed a 22-year period from 1999 through 2020, focusing on mortality trends associated with bladder malignancies among adults 25 years of age and older. We identified relevant cases using the International Classification of Diseases, 10th Revision (ICD-10) code C67, which specifically designates bladder malignancies. Throughout our analysis, we adhered rigorously to the Strengthening the Reporting of Observational Studies in Epidemiology reporting guidelines (22).

The analytical framework incorporated multiple demographic and geographic variables. Demographic parameters included sex (categorized as male or female), race/ethnicity (classified as Hispanic or Latino, Black or African American, and White populations), and age (stratified into seven distinct groups: 25-34, 35-44, 45-54, 55-64, 65-74, 75-84, and ≥85 years). Geographic variables comprised urbanization level (categorized as Metropolitan or Nonmetropolitan according to the 2013 US Census classification), state of residence, and broader census region (divided into four major areas as defined by the US Census Bureau: Northeast, Midwest, South, and West).From the database, we extracted comprehensive annual mortality metrics including absolute number of deaths, crude mortality rates, and age-adjusted mortality rates (AAMR, standardized to the 2000 US standard population), along with their corresponding 95% confidence intervals (CI).

### 2.3 Statistical analysis

For the entire study period and across all stratified conditions, we calculated comprehensive mortality metrics including death counts, proportional composition, average annual crude mortality rates, and age-adjusted mortality rates (AAMRs). To facilitate nuanced analysis, we stratified data by year, sex, race/ethnicity, state, and urbanization status, creating distinct analytical cohorts. All statistical estimates were presented with corresponding 95% confidence intervals (CIs). Crude mortality rates were derived by dividing bladder cancer-related deaths in each relevant year by the corresponding US population size. For standardization purposes, AAMRs were calculated using the 2000 US standard population as the reference.

For trend analysis, we employed Joinpoint regression software (Version 4.9.1.0) developed by the National Cancer Institute. This sophisticated modeling approach identifies statistically significant inflection points (joinpoints) in temporal mortality trends through rigorous Monte Carlo permutation tests, effectively segmenting the complete time series into consecutive linear components. For each identified segment, we calculated the annual percent change (APC) with corresponding 95% CIs. Additionally, we summarized the overall trend across the entire study period using the average annual percent change (AAPC) with its 95% CI. Statistical significance was established at P < 0.05 (two-tailed).The Joinpoint regression algorithm initiates with a simple single-line model and sequentially evaluates whether additional joinpoints significantly enhance model fit. Importantly, we determined the optimal segmentation structure independently for each subgroup, allowing for distinct temporal patterns across demographic and geographic categories rather than imposing a uniform structure. This methodological approach enabled more precise calculation of segment-specific APCs and their corresponding confidence intervals for each identified interval (23).

## 3 Results

### 3.1 Overall mortality trends

From 1999 to 2020, a total of 319,229 adults aged 25 years and older died from bladder cancer in the United States. The overall AAMR was 6.686 per 100,000 population (95% CI: 6.663-6.709). Among these deaths, 224,737 (70.40%) occurred in males and 94,492 (29.60%) in females. By race/ethnicity, 15,532 deaths occurred among Hispanic or Latino individuals, 23,648 among Black or African American individuals, and 290,049 among White individuals. Age distribution revealed that 2,306 deaths (0.72%) occurred in young adults aged 25-44 years, 46,442 deaths (14.55%) in middle-aged adults aged 45-64 years, and 270,481 deaths (84.73%) in older adults aged 65 years and above (**Table 1** and **Supplementary Table S1**). Among the 319229 deaths with recorded place of death,25.49% occurred in medical facilities,19.43% in nursing homes/long-term care facilities,8.77% in hospice facilities, and 40.42% in the decedent’s home (**Supplementary Tables S2, S3**).

**Table 1:** Demographic characteristics of deaths due to bladder cancer in the United States from 1999 to 2020.

Variables	Deaths	Population	AAMR(95%CI)
Overall	319229	4473854489	6.686(6.663-6.709)
Sex			
Male	224737	2154556911	11.574(11.525-11.623)
Female	94492	2319297578	3.382(3.360-3.404)
Race/ethnicity			
Hispanic or Latino	5532	295429555	5.544(5.421-5.820)
Black or African American	23648	546447527	5.482(5.411-5.553)
White	290049	3631977407	7.003(6.977-7.028)
Census region			
Northeast	68056	827193779	7.191(7.137-7.245)
Midwest	74983	969567311	6.933(6.883-6.983)
South	111913	1652256217	6.478(6.440-6.516)
West	64277	1024837182	6.353(6.304-6.402)
Urbanization			
Metropolitan(Urban)	261046	3795213822	6.64(6.614-6.665)
Nonmetropolitan(Rural)	58183	678634169	6.887(6.831-6.944)
Ten-year age groups			
25-34 years	293	920089469	0.032(0.028-0.035)
35-44 years	2013	931287288	0.216(0.207-0.226)
45-54 years	10996	927576220	1.185(1.163-1.208)
55-64 years	35446	766424847	4.625(4.557-4.673)
65-74 years	70137	510458341	13.74(13.638-13.842)
75-84 years	108568	298504433	36.371(36.154-36.587)
85+ years	91776	119513891	76.791(76.294-77.288)
Place of death			
Medical Facility	81360	–	–
Decedent’s home	129039	–	–
Hospice facility	27990	–	–
Nursing home/long-term care	62032	–	–
Other	18005	–	–
Place of death unknown	803	–	–

AAMR, age‐adjusted mortality rate; CI, confidence interval.

Joinpoint regression analysis revealed a significant nonlinear declining trend (overall AAPC = -0.410, 95% CI: -0.495 to -0.310; P < 0.001). Specifically, mortality rates demonstrated a slow upward trend from 1999 to 2010 (APC = 0.184, 95% CI: 0.054 to 1.087; P = 0.016), followed by a gradual decline from 2010 to 2016 (APC = -0.347, 95% CI: -1.095 to 0.006; P = 0.054), and subsequently showed a significantly accelerated decline from 2016 to 2020 (APC = -2.118, 95% CI: -3.022 to -1.582; P < 0.001) (**Table 2**, **Supplementary Figure S1**).

**Table 2:** Annual percentage changes and average annual percentage changes in bladder cancer in the USA from 1999 to 2020.

Variables	Trend segment	Year interval	APC (95% CI)	AAPC (95% CI)	*P* value
Entire cohort	–	1999-2020	–	-0.410(-0.495 to -0.310)	<0.001
	1	1999-2010	0.184(0.054 to 1.087)	–	0.016
	2	2010-2016	-0.347(-1.095 to 0.006)	–	0.054
	3	2016-2020	-2.118(-3.022 to -1.582)	–	<0.001
Sex					
Male	–	1999-2020	–	-0.516(-0.615 to -0.401)	<0.001
	1	1999-2010	0.149(-0.008 to 1.058)	–	0.057
	2	2010-2016	-0.598(-1.131 to -0.079)	–	0.024
	3	2016-2020	-2.198(-3.290 to -1.612)	–	<0.001
Female	–	1999-2020	–	-0.888(-1.127 to -0.652)	<0.001
	1	1999-2017	-0.510(-0.665 to -0.162)	–	0.036
	2	2017-2020	-3.130(-5.843 to 1.129)	–	<0.001
Race/ethnicity					
Hispanic or latino	–	1999-2020	–	-0.337(-0.799 to 0.303)	0.329
Black or African American	–	1999-2020	–	-1.138(-1.555 to -0.468)	0.002
	1	1999-2017	-0.723(-1.111 to 4.407)	–	0.133
	2	2017-2020	-3.93(-8.822 to -0.86	–	0.001
White	–	1999-2020	–	-0.254(-0.331 to -0.173)	<0.001
	1	1999-2010	0.360(0.236 to 0.822)	–	0.002
	2	2010-2016	-0.200(-0.832 to 0.133)	–	0.220
	3	2016-2020	-2.002(-2.795 to -1.512)	–	<0.001
Census region					
Northeast	–	1999-2020	–	-1.025(-1.211 to -0.857)	<0.001
	1	1999-2016	-0.224(-0.400 to -0.021)	–	0.029
	2	2016-2020	-4.358(-6.385 to -3.054)	–	<0.001
Midwest	–	1999-2020	–	-0.281(-0.420 to -0.144)	<0.001
	1	1999-2013	0.274(0.074 to 0.535)	–	0.007
	2	2013-2020	-1.380(-2.129 to -0.879)	–	<0.001
South	–	1999-2020	–	-0.118(-0.280 to 0.035)	0.116
	1	1999-2015	0.276(0.114 to 0.524)	–	0.001
	2	2015-2020	-1.368(-2.840 to -0.632)	–	<0.001
West	–	1999-2020	–	-0.444(-0.653 to -0.193)	<0.001
	1	1999-2013	-0.051(-0.301 to 0.882)	–	0.909
	2	2013-2020	-1.226(-3.061 to -0.585)	–	<0.001
Urbanization					
Metropolitan(Urban)	–	1999-2020	–	-0.616(-0.743 to -0.505)	<0.001
	1	1999-2016	-0.122(-0.239 to 0.027)	–	0.100
	2	2016-2020	-2.687(-3.940 to -1.840)	–	<0.001
Nonmetropolitan(Rural)	–	1999-2020	–	0.269(0.063 to 0.506)	0.024
	1	1999-2011	0.836(0.513 to 1.621)	–	<0.001
	2	2011-2020	-0.483(-1.500 to -0.011)	–	0.047

### 3.2 Trends in mortality stratified by sex

**Figure 1:**
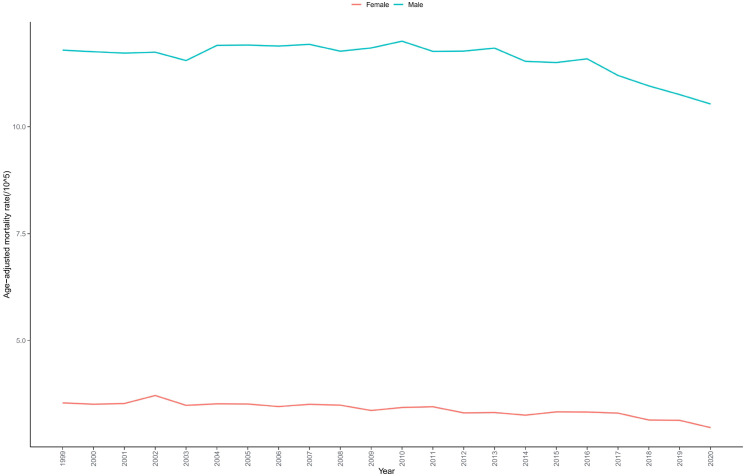
Trends in bladder cancer-related mortality in US adults stratified by sex from 1999 to 2020.

Male bladder cancer mortality rates (AAMR = 11.574 per 100,000) were significantly higher than female rates (AAMR = 3.382 per 100,000) (**Table 1**, **Supplementary Table S4**). Both sexes exhibited declining mortality trends, but with distinct temporal patterns. Male mortality rates showed an upward trend from 1999-2010 (APC = 0.149), began a gradual decline from 2010-2016 (APC = -0.598), and demonstrated a rapid decline from 2016-2020 (APC = -2.198) (overall AAPC = -0.516). In contrast, female mortality rates exhibited a gradual decline from 1999-2017 (APC = -0.510), followed by an accelerated decline from 2017-2020 (APC = -3.130) (overall AAPC = -0.888). Consequently, the absolute mortality gap between males and females widened during the study period (**Table 2**, **Figure 1**).

### 3.3 Mortality trends stratified by race and ethnicity

**Figure 2:**
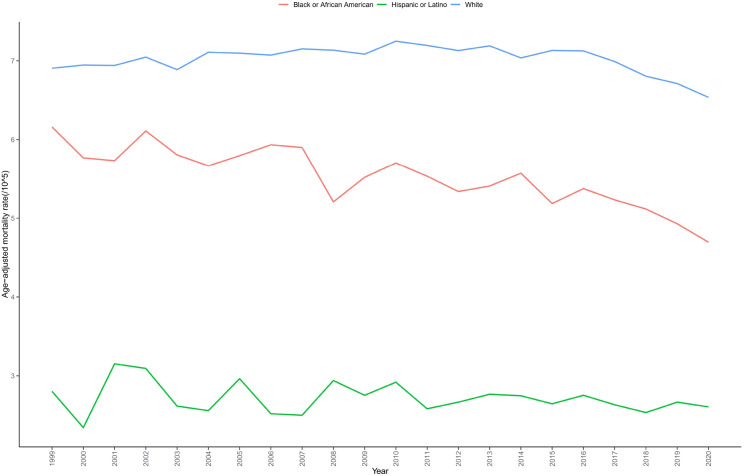
Trends in bladder cancer-related mortality in US adults stratified by race from 1999 to 2020.

White individuals showed the highest recorded AAMR (7.003 per 100,000), followed by Hispanic or Latino individuals (5.544 per 100,000), while Black or African American individuals had the lowest recorded mortality rate (5.482 per 100,000) (**Table 1**, **Supplementary Table S5**). However, it is important to note that these observed differences may reflect systematic diagnostic and coding biases rather than true biological disparities. Death certificate data may underestimate bladder cancer mortality in racial and ethnic minorities due to potential attribution of deaths to secondary complications rather than accurately identifying bladder cancer as the underlying cause (a “diagnostic dilution effect”). All racial/ethnic groups demonstrated declining mortality trends, but the magnitude of decline varied considerably. Black or African American individuals experienced the most substantial decline (AAPC=-1.138), while White individuals demonstrated the smallest reduction (AAPC=-0.254), with rates remaining stable during 2012-2016 (APC=-0.200, P = 0.220) followed by a rapid decline during 2016-2020 (APC=-2.002). Hispanic or Latino mortality rates remained relatively stable without major fluctuations, exhibiting a consistent modest decline overall (AAPC=-0.337, P = 0.329), though this trend was not statistically significant (**Table 2**, **Figure 2**).

### 3.4 Mortality trends stratified by urbanization level

**Figure 3:**
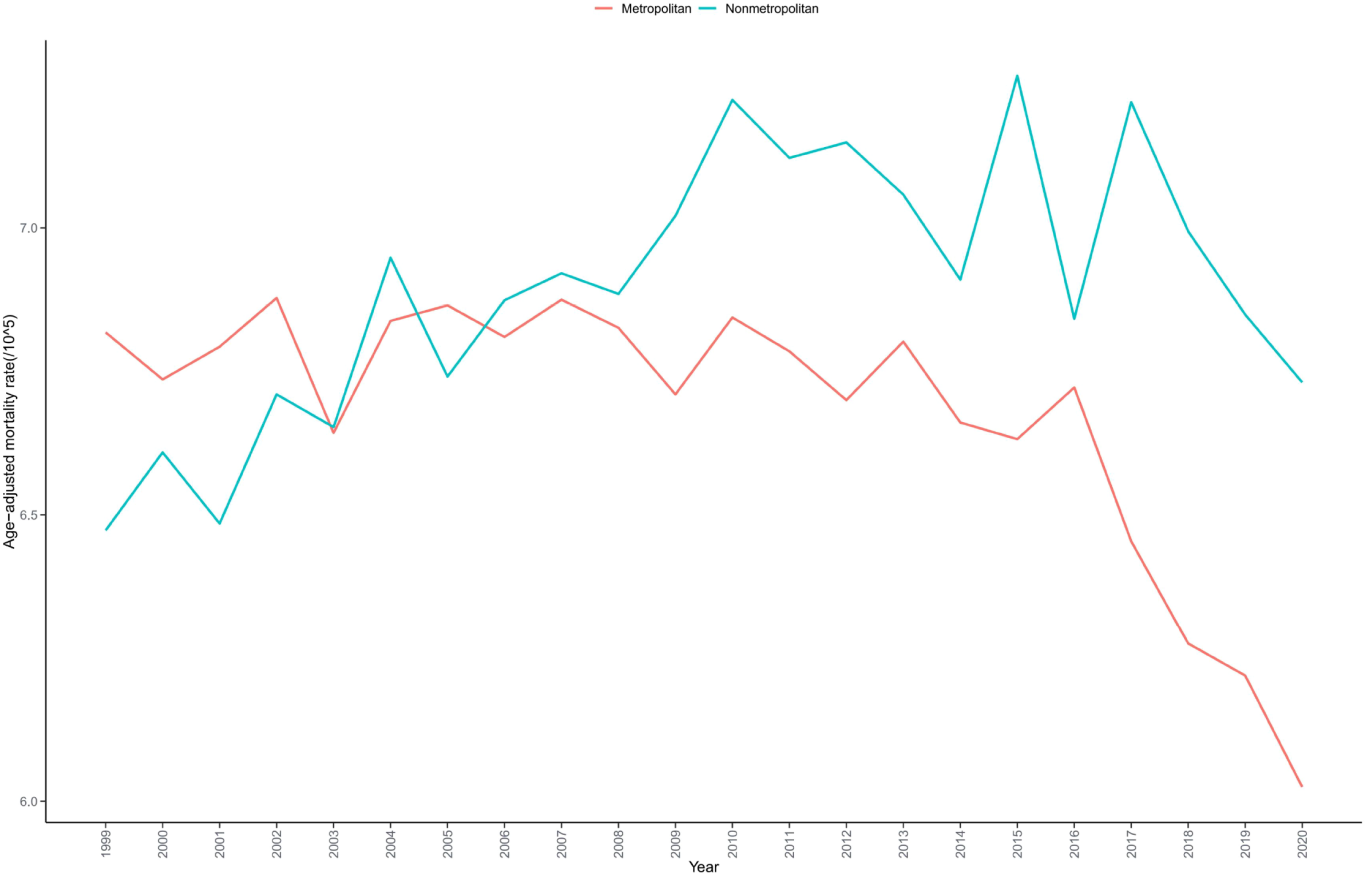
Trends in bladder cancer-related mortality in US adults stratified by urban-rural classification from 1999 to 2020.

Residents of nonmetropolitan areas had higher bladder cancer mortality rates (AAMR = 6.887 per 100,000) compared to residents of metropolitan areas (AAMR = 6.640 per 100,000) (**Table 1**, **Supplementary Table S6**). Nonmetropolitan areas exhibited an increasing mortality trend (AAPC = 0.269), while metropolitan areas demonstrated a declining trend (AAPC = -0.616), indicating significant geographic disparities in bladder cancer mortality rates between nonmetropolitan and metropolitan regions (**Table 2**, **Figure 3**).

### 3.5 Mortality trends stratified by region

**Figure 4:**
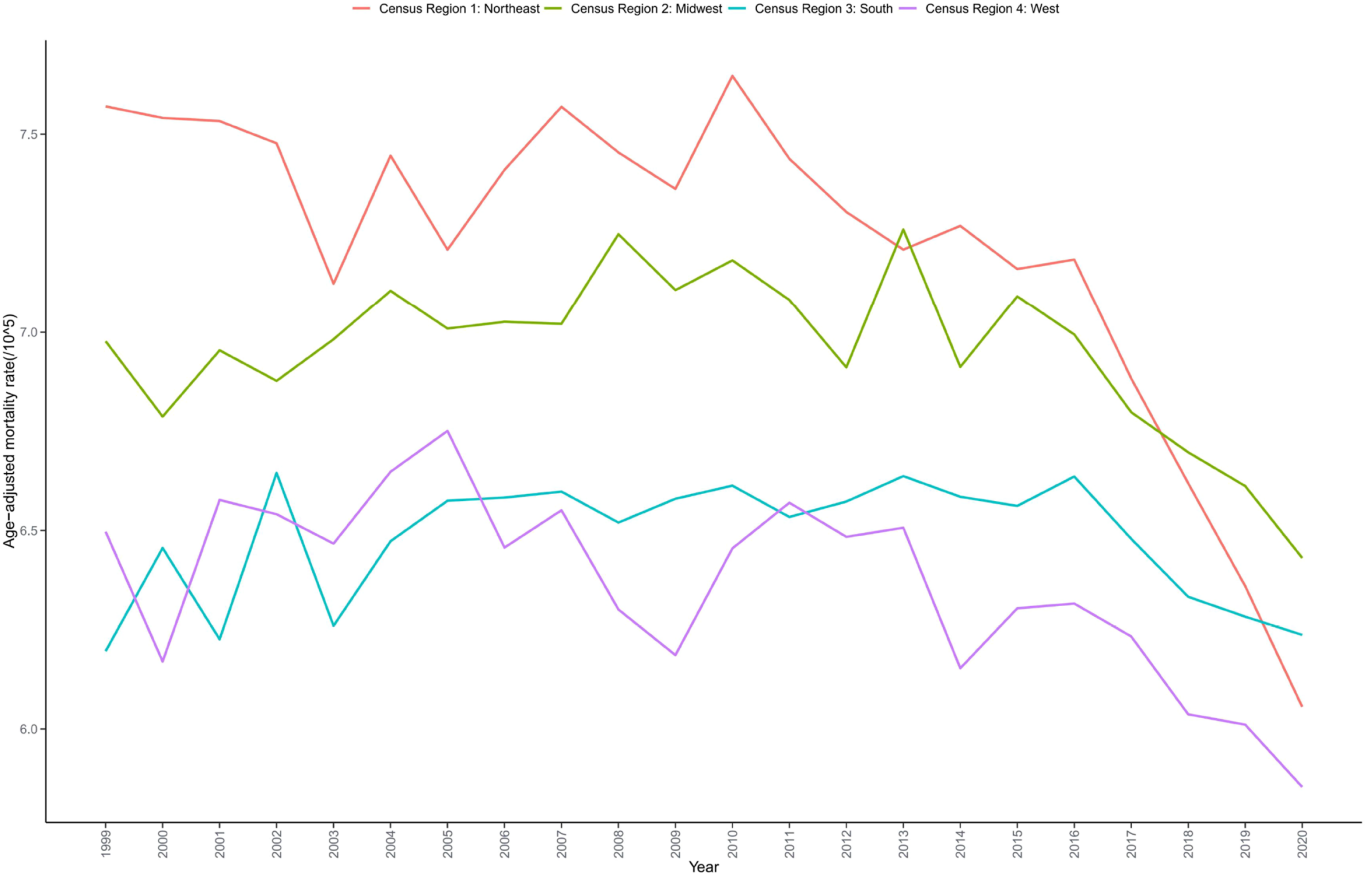
Trends in bladder cancer-related mortality in US adults stratified by census region from 1999 to 2020.

The Northeast region had the highest AAMR (7.191 per 100,000), followed by the Midwest (6.933 per 100,000), South (6.478 per 100,000), and West (6.353 per 100,000) (**Table 1**, **Supplementary Table S7**). All four regions demonstrated declining trends, with three regions showing statistically significant decreases (P < 0.05). However, the South exhibited the smallest decline (AAPC = -0.118; P = 0.116), which was not statistically significant, while the Northeast demonstrated the largest decline (AAPC = -1.025; P < 0.001) (**Table 2**, **Figure 4**).

### 3.6 Mortality trends stratified by state

Significant variations in bladder cancer-related mortality rates were observed across US states. Maine had the highest AAMR (8.868; 95% CI: 8.495-9.242), followed by Vermont (8.229; 95% CI: 7.682-8.776), Nevada (7.983; 95% CI: 7.683-8.282), Delaware (7.937; 95% CI: 7.479-8.394), and New Hampshire (7.835; 95% CI: 7.457-8.213). These states are predominantly located in the Northeast and West regions.

**Figure 5:**
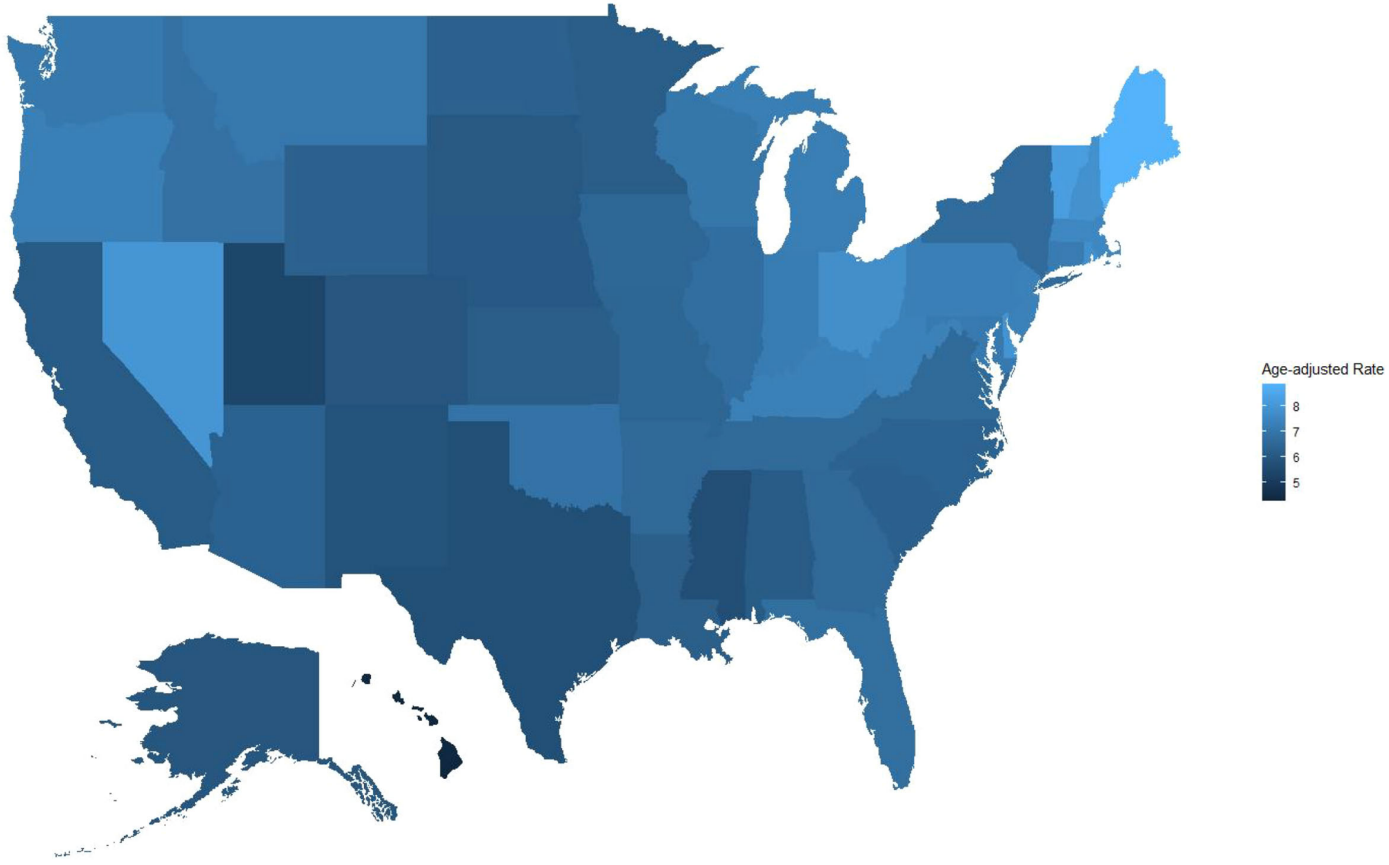
AAMRs associated with bladder cancer deaths per 100,000 population in US states from 1999 to 2020.

In contrast, Hawaii had the lowest AAMR (4.292; 95% CI: 4.025-4.560), followed by Utah (5.428; 95% CI: 5.158-5.699) and Mississippi (5.676; 95% CI: 5.450-5.907). These geographic disparities may reflect differences in healthcare accessibility, underdiagnosis of bladder malignancies, or variations in death certificate coding practices. Overall, the interstate variations highlight potential inequities in disease recognition and provide opportunities for targeted educational, diagnostic, and policy interventions (**Supplementary Table S8** and **Figure 5**).

### 3.7 Mortality stratified by age

bladder cancer-related mortality rates demonstrated an exponential increase with advancing age. The youngest age group (25-34 years) had the lowest AAMR of 0.032 per 100,000 (95% CI: 0.028-0.035), while the oldest age group (≥85 years) had an AAMR of 76.791 per 100,000 (95% CI: 76.294-77.288) (**Table 1**).

## 4 Discussion

Drawing upon nationally representative mortality data, this study systematically illuminates the complex evolutionary trajectory of adult bladder cancer mortality across the United States from 1999 through 2020. Our principal findings reveal that despite an overall modest decline in mortality rates (AAPC: -0.410%), this trend exhibits distinctive phase-specific characteristics: an initial period of slight increase (1999–2010), followed by an intermediate phase of relative stability (2010–2016), culminating in a subsequent period of significant decline (2016-2020, APC: -2.118%). This nonlinear pattern of temporal variation, coupled with persistent and substantial disparities across sex, race, geographic regions, and urbanization levels, offers critical insights into both the dynamic evolution of bladder cancer disease burden and the deeply entrenched health inequities that permeate the healthcare system (24).

The triphasic evolution of overall mortality reflects major transformations in the bladder cancer diagnosis and treatment landscape. The significant decline after 2016 can be largely attributed to the introduction and widespread adoption of immune checkpoint inhibitors. Mechanistically, these agents function by disrupting the PD-1/PD-L1 signaling axis that tumor cells exploit to evade immune surveillance, thereby restoring cytotoxic T-cell activity against bladder cancer cells (14). This approach proved particularly effective in bladder cancer due to its high mutational burden and consequent neoantigen expression, creating an immunologically “hot” tumor microenvironment responsive to immunotherapeutic intervention. Pembrolizumab, approved by the FDA in 2017 as second-line therapy for locally advanced or metastatic urothelial carcinoma following platinum-based chemotherapy progression, demonstrated in pivotal clinical trials a median overall survival extension of approximately 3 months compared to chemotherapy (10.1 months vs. 7.3 months, HR = 0.70, 95% CI: [missing from original]) (14). Subsequently, the successive approvals of other PD-L1 inhibitors such as Atezolizumab, along with the gradual implementation of these agents in first-line treatment, significantly improved survival outcomes for patients with advanced bladder cancer (25). Notably, the benefits of immunotherapy were not confined to clinical trial populations, as real-world studies demonstrated similar survival improvements across broad clinical practice settings (26). The therapeutic impact was amplified by concurrent advances in molecular profiling techniques, enabling better patient selection through PD-L1 expression assessment and genomic instability markers, which likely contributed to optimizing treatment allocation and enhancing population-level mortality benefits.

However, it is important to acknowledge that the observed mortality decline after 2016 may reflect a complex interplay of factors beyond therapeutic advances alone. Improvements in diagnostic technology and screening practices during this period may have contributed to earlier case detection, creating lead-time bias that artificially enhances apparent survival outcomes. Additionally, changes in diagnostic intensity including the widespread adoption of enhanced imaging modalities and molecular profiling techniques may have altered the composition of the diagnosed population in ways that influence mortality statistics. While we attribute the primary driver of improved outcomes to immunotherapy based on the temporal alignment with FDA approvals and clinical trial evidence, the multifactorial nature of cancer care improvements makes it challenging to isolate the specific contribution of any single intervention.

However, the slight upward trend from 1999 to 2010 warrants careful consideration. This period coincided with accelerated population aging in the United States, and bladder cancer, as an age-related malignancy, exhibits exponentially increasing risk with age evidenced by the extremely high mortality rate (76.791 per 100,000) in the ≥85 years age group observed in this study (17). Demographic shifts may have partially offset the benefits derived from early advances in diagnostic and therapeutic technologies. Additionally, smoking-related bladder cancer deaths during this period may not yet have reflected the long-term effects of tobacco control policies, given that the latency period for smoking-induced carcinogenesis typically spans 15-30 years (9).

The significant sex disparities revealed in this study (male-to-female mortality ratio of approximately 3.4:1) substantially exceed the sex ratio in incidence rates (approximately 3:1), suggesting that female patients may face more complex challenges (5). The mechanisms underlying this phenomenon are likely multifaceted and operate at biological, behavioral, and healthcare system levels. First, the role of sex bias in primary care cannot be overlooked – when women present with typical symptoms such as hematuria, clinicians are more likely to initially consider benign conditions such as urinary tract infections, leading to delayed referral and diagnostic delays (27). This diagnostic delay mechanism is compounded by sex-based differences in symptom reporting patterns, as women may describe bladder symptoms differently than the classical presentations emphasized in medical education (27). Second, female bladder cancer patients are often diagnosed at more advanced stages, which reflects both diagnostic delays and fundamental biological differences (28). Recent molecular research indicates sex-specific tumor biology, with differential expression of steroid hormone receptors (particularly estrogen receptor β and androgen receptors) influencing tumor aggressiveness and treatment response (28). The protective effect of estrogen may diminish after menopause, potentially explaining the higher proportion of aggressive variants in elderly female patients. Furthermore, pharmacokinetic and pharmacodynamic differences between sexes affect drug metabolism, with women experiencing both different toxicity profiles and efficacy outcomes from standard chemotherapeutic regimens (29). Finally, women have historically lower rates of receiving radical treatments such as radical cystectomy, which may impact long-term survival (29). This treatment disparity mechanism operates through both physician bias in treatment recommendations and sex-based differences in treatment preferences and risk tolerance, creating a complex web of factors that collectively drive the observed mortality gap.

The analysis of racial disparities revealed a seemingly paradoxical yet clinically significant finding: non-Hispanic White individuals had the highest recorded mortality rate (7.003 per 100,000), while non-Hispanic Black individuals had relatively lower recorded mortality rates (5.482 per 100,000). However, this finding requires careful interpretation, as substantial literature suggests it most likely reflects systematic diagnostic and coding biases rather than true biological differences in disease burden (30).Multiple studies have documented significant racial disparities in death certificate coding accuracy that support our interpretation. Singh and Siahpush (31) demonstrated that cancer deaths among African Americans are systematically under-reported on death certificates, with cancer mortality rates being underestimated by 5-15% compared to whites due to preferential attribution to cardiovascular or infectious complications. Similarly, Arias et al. (32) found that race-specific differences in death certificate completion practices result in systematic misclassification, where minority populations’ deaths are more frequently coded to immediate rather than underlying causes. This “diagnostic dilution effect” operates through multiple mechanisms. Racial and ethnic minorities, particularly Black populations, have long faced barriers to healthcare access, inadequate insurance coverage, and disparities in care quality (31, 33). As a result, their bladder cancer deaths are more likely to be attributed to secondary complications (such as cardiac or renal failure, septic shock) rather than being accurately identified and coded as bladder cancer-related deaths. Coresh et al. (34) found that among patients with confirmed genitourinary malignancies at autopsy, Black patients were 35% less likely than White patients to have the cancer listed as the underlying cause of death, even when tumor burden was equivalent.

Furthermore, disparities in access to subspecialty care mean that minority patients may die without definitive cancer staging or histologic confirmation, leading to death certificate coding based on clinical presentation rather than established cancer diagnosis (35). Williams et al. (18) specifically documented in bladder cancer that Black patients experience significant delays in specialist referral and are more likely to receive palliative rather than curative intent care, factors that may contribute to death certificate misclassification. The raw mortality rates presented in this study should therefore not be interpreted as evidence that White individuals bear a higher true burden of bladder cancer mortality. Notably, the significant decline in recorded mortality rates among Black populations (AAPC: -1.138%) may reflect the positive effects of recent healthcare insurance expansion (such as the Affordable Care Act) and policy measures aimed at reducing health disparities, potentially improving both access to care and accuracy of diagnostic coding (36).

Our analysis of place of death revealed that 40.42% of bladder cancer deaths occurred at home, followed by 28.27% in inpatient facilities,17.12% in nursing homes/long-term care facilities, and 9.14% in hospice facilities. The substantial proportion of home deaths highlights the importance of adequate home-based palliative care services for bladder cancer patients. This finding aligns with the national trend toward home-based end-of-life care and reflects both patient preferences for dying at home and the expanding availability of home hospice services (37). However, it also underscores the need for robust support systems for family caregivers, who shoulder significant responsibilities in home-based end-of-life care. The relatively high proportion of deaths in nursing homes(17.12%) further reflects the age distribution of bladder cancer mortality, as many elderly patients with bladder cancer may already reside in long-term care facilities. Healthcare systems should ensure that these facilities are adequately equipped to provide appropriate palliative care services for bladder cancer patients.

Geographic disparities represent one of the most concerning findings in this study. Rural areas not only had higher mortality rates than urban areas but, more critically, exhibited completely opposite trends rural areas showed an increasing trend (AAPC: +0.269%), while urban areas demonstrated a declining trend (AAPC: -0.616%). This “urban-rural divide” reflects structural inequalities operating through multiple mechanistic pathways in the healthcare ecosystem (37). The specialist density mechanism is particularly relevant for bladder cancer, where diagnosis and treatment heavily rely on subspecialty expertise rural areas face critical shortages of urologists (with some counties having no urologists at all), medical oncologists, and radiation oncologists, creating compounding barriers to guideline-concordant care (37). This workforce deficit operates alongside a technology access mechanism, where advanced diagnostic tools essential for accurate staging (such as enhanced cystoscopy technologies, specialized pathology services, and high-resolution imaging) are concentrated in urban academic centers (38). The care coordination mechanism further exacerbates disparities, as rural health systems often lack the integrated electronic health records and formalized referral networks necessary for timely transitions between primary care, specialist evaluation, and tertiary care centers (38). At the facility level, volume-outcome relationships in bladder cancer surgery create a quality gap, with rural patients frequently receiving care at low-volume centers associated with higher complication rates and inferior oncologic outcomes (39). Research by Deuker et al. demonstrated that rural patients experience significant delays in bladder cancer diagnosis and substantial gaps in accessing curative treatments (39). These delays activate biological mechanisms where tumor progression during diagnostic and treatment intervals leads to stage migration, reducing treatment effectiveness. More importantly, as medical technology becomes increasingly complex and specialized, the healthcare quality gap between urban and rural areas tends to widen further through a technological diffusion mechanism, where innovations like immunotherapy and molecular testing are implemented first in academic centers and disseminate slowly to community practices, particularly those in rural settings.

State-level analysis further supported the importance of geographic factors. Northeastern states such as Maine (8.868 per 100,000) and Vermont (8.229 per 100,000) had the highest mortality rates. Common characteristics of these regions include: severe population aging, extensive rural areas, and relatively scarce specialty medical resources. In contrast, the low mortality rates in Hawaii (4.292 per 100,000) and Utah (5.428 per 100,000) may be related to their unique population structures (relatively young, health-conscious) and lifestyle factors (40).

Age-stratified results emphasized the characteristics of bladder cancer as an age-related disease, with 84.73% of deaths occurring in individuals ≥65 years old. High mortality rates in elderly patients reflect both intrinsic biological mechanisms and healthcare delivery factors. At the cellular level, age-related accumulation of DNA damage, particularly from environmental carcinogens like tobacco smoke, drives bladder carcinogenesis through specific mutational signatures (41). The process of immunosenescence progressive deterioration of immune system function with aging further contributes to both increased cancer susceptibility and reduced immunotherapy efficacy in elderly patients (41).

Analysis of place of death provided additional insights. 42.95% of bladder cancer patients died at home, a proportion higher than most other cancers, possibly reflecting that late-stage bladder cancer patients more frequently choose comfort care over aggressive treatment, highlighting the importance of hospice care services (42).

Study limitations include several important considerations. First, death certificate data has inherent limitations including misclassification of underlying causes of death and coding biases that may not fully reflect true bladder cancer disease burden. This is particularly relevant for racial disparities, as our finding of higher mortality rates among White individuals likely reflects coding biases rather than biological differences. The “diagnostic dilution effect” – where minority population deaths are more frequently attributed to secondary complications rather than bladder cancer may mask true health inequities. Second, survivorship bias may impact our findings, as improved case detection and earlier diagnosis over time can create apparent mortality improvements through lead-time bias rather than genuine therapeutic advances. Enhanced diagnostic technologies including improved cystoscopy and imaging may identify more early-stage, treatable cases, artificially improving mortality trends. Third, confounding by changes in diagnostic intensity represents a significant limitation. Over the study period, widespread adoption of advanced imaging, molecular profiling, and systematic screening may have altered the diagnosed population composition, affecting mortality calculations in ways that are difficult to quantify without concurrent incidence data. Fourth, the lack of individual-level covariates such as smoking history, tumor staging, treatment details, and comorbidity status limits causal inferences about observed trends. Finally, the CDC WONDER database cannot distinguish whether mortality declines reflect reduced incidence (prevention success) or improved survival (treatment effectiveness), limiting our understanding of the mechanistic drivers behind observed trends.

In conclusion, this study revealed the complex transformations in bladder cancer mortality in the United States over the past two decades. While the overall trend shows improvement, persistent deep-rooted health inequalities cannot be ignored. Future public health interventions and clinical practice must adopt precision-based, individualized strategies, with particular attention to high-risk populations (rural residents, elderly patients, certain racial groups) and regions. Through policy innovation, resource reallocation, and technological approaches, efforts must be made to eliminate these disparities and achieve health equity goals in bladder cancer prevention and control.

## Data Availability

The original contributions presented in the study are included in the article/**Supplementary Material**. Further inquiries can be directed to the corresponding author.
