# Supplementary material for: Evolving patterns of bladder cancer mortality in US adults: a two-decade analysis reveals persistent disparities despite overall progress (1999-2020)
Source: Front Oncol. 2026 Mar 2;16:1713506. doi: 10.3389/fonc.2026.1713506 (PMC12989382; doi:10.3389/fonc.2026.1713506)
Supplement: Supplementary file 1 [file DataSheet1.doc]

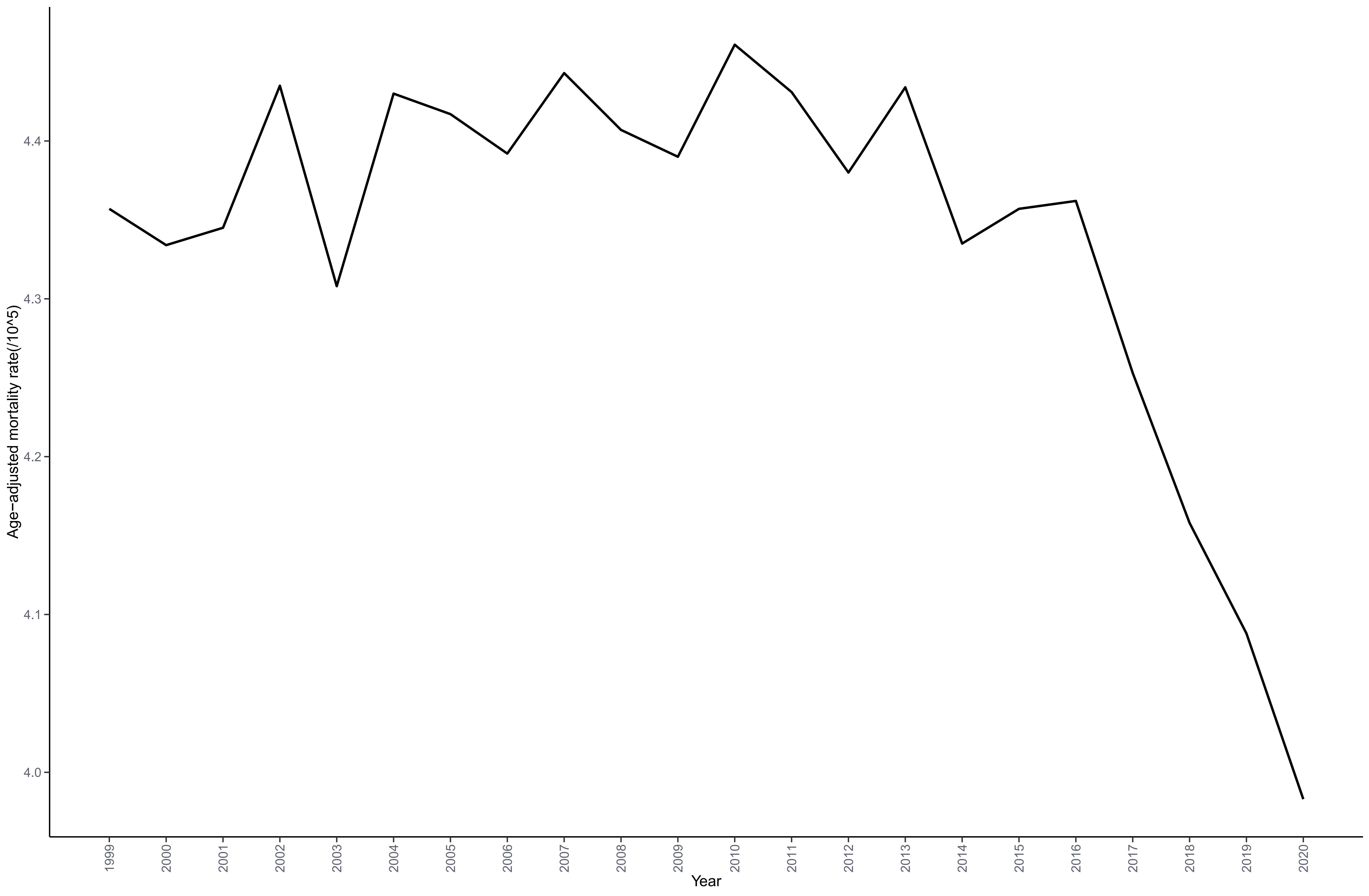


**Supplementary Figure S1.Trends in bladder cancer-related mortality among adults in the United States from 1999 to 2020.**

**Supplementary Table S1. Bladder cancer-related mortality in US adults overall and by sex and ethnic stratification from 1999 to 2020.**

|  | **Overall** | **Female** | **Male** | **Hispanic or Latino** | **Black or African American** | **White people** | **Population** |
| --- | --- | --- | --- | --- | --- | --- | --- |
| **1999** | 11908 | 3805 | 8103 | 130 | 917 | 10861 | 180408769 |
| **2000** | 11998 | 3838 | 8160 | 114 | 851 | 11033 | 181984640 |
| **2001** | 12221 | 3881 | 8340 | 168 | 877 | 11176 | 184305128 |
| **2002** | 12623 | 4121 | 8502 | 175 | 940 | 11508 | 186208028 |
| **2003** | 12482 | 3907 | 8575 | 160 | 914 | 11408 | 188090429 |
| **2004** | 13028 | 4035 | 8993 | 160 | 912 | 11956 | 190205384 |
| **2005** | 13253 | 4063 | 9190 | 192 | 961 | 12100 | 192551384 |
| **2006** | 13473 | 4062 | 9411 | 181 | 1007 | 12285 | 195019359 |
| **2007** | 13842 | 4199 | 9643 | 189 | 1033 | 12620 | 197403777 |
| **2008** | 14032 | 4245 | 9787 | 233 | 949 | 12850 | 199795090 |
| **2009** | 14198 | 4158 | 10040 | 228 | 1025 | 12945 | 202107016 |
| **2010** | 14730 | 4302 | 10428 | 254 | 1073 | 13403 | 203891983 |
| **2011** | 15007 | 4415 | 10592 | 242 | 1109 | 13656 | 206592936 |
| **2012** | 15239 | 4356 | 10883 | 265 | 1090 | 13884 | 208826037 |
| **2013** | 15756 | 4463 | 11293 | 296 | 1167 | 14293 | 211085314 |
| **2014** | 15773 | 4483 | 11290 | 317 | 1227 | 14229 | 213809280 |
| **2015** | 16252 | 4665 | 11587 | 322 | 1191 | 14739 | 216553817 |
| **2016** | 16644 | 4705 | 11939 | 353 | 1266 | 15025 | 218641417 |
| **2017** | 16655 | 4769 | 11886 | 357 | 1276 | 15022 | 221447331 |
| **2018** | 16640 | 4663 | 11977 | 366 | 1305 | 14969 | 223311190 |
| **2019** | 16794 | 4739 | 12055 | 412 | 1277 | 15105 | 224981167 |
| **2020** | 16681 | 4618 | 12063 | 418 | 1281 | 14982 | 226635013 |
| **Total** | 319229 | 94492 | 224737 | 5532 | 23648 | 290049 | 4473854489 |

**Supplementary Table S2. Bladder cancer-related mortality in US adults stratified by place of death from 1999 to 2020.**

|  | **Medical Facility** | **Nursing home/Long Term Care** | **Hospice facility** | **Decedent's home** | **Other** | **Place of death unknown** |
| --- | --- | --- | --- | --- | --- | --- |
| **1999** | 4403 | 2648 | Missing | 4241 | 614 | Missing |
| **2000** | 4152 | 2788 | Missing | 4385 | 671 | Missing |
| **2001** | 4281 | 2751 | Missing | 4439 | 750 | 0 |
| **2002** | 4264 | 2825 | Missing | 4626 | 907 | Missing |
| **2003** | 4105 | 2782 | 86 | 4598 | 872 | 39 |
| **2004** | 3993 | 2896 | 171 | 4900 | 1039 | 29 |
| **2005** | 3950 | 2906 | 467 | 5060 | 844 | 26 |
| **2006** | 4023 | 2838 | 602 | 5108 | 863 | 39 |
| **2007** | 4054 | 2867 | 862 | 5214 | 826 | 19 |
| **2008** | 3795 | 2877 | 1079 | 5260 | 783 | 238 |
| **2009** | 3552 | 2950 | 1081 | 5388 | 870 | 349 |
| **2010** | 3682 | 3027 | 1372 | 5774 | 870 | Missing |
| **2011** | 3519 | 2898 | 1582 | 6090 | 912 | Missing |
| **2012** | 3403 | 2743 | 1901 | 6252 | 932 | Missing |
| **2013** | 3322 | 2782 | 1934 | 6661 | 1051 | Missing |
| **2014** | 3273 | 2791 | 2280 | 6658 | 755 | 16 |
| **2015** | 3282 | 2920 | 2411 | 6943 | 694 | Missing |
| **2016** | 3374 | 2898 | 2549 | 7124 | 695 | Missing |
| **2017** | 3420 | 2872 | 2516 | 7063 | 771 | Missing |
| **2018** | 3400 | 2784 | 2515 | 7181 | 751 | 0 |
| **2019** | 3263 | 2913 | 2546 | 7366 | 703 | Missing |
| **2020** | 2823 | 2276 | 2036 | 8708 | 832 | Missing |
| **Total** | 81360 | 62032 | 27990 | 129039 | 18005 | 803 |

**Supplementary Table S3. Bladder cancer-related mortality in US adults stratified by place of death from 1999 to 2020.**

| **Death Location** | **Number of deaths** | **Percent** |
| --- | --- | --- |
| **Medical Facility** | 81360 | 25.49% |
| **Decedent's home** | 129039 | 40.42% |
| **Hospice facility** | 27990 | 8.77% |
| **Nursing home/long-term care** | 62032 | 19.43% |
| **Other** | 18005 | 5.64% |
| **Place of death unknown** | 803 | 0.25% |
| **Total** | 319229 | 100% |

**Supplementary Table S4.Bladder cancer-associated AAMR overall and sex-stratified per 100,000 people in the United States 1999-2020.**

| **Year** | **Overall** | **Female** | **Male** |
| --- | --- | --- | --- |
| **1999** | 6.737(6.616-6.858) | 3.539(3.426-3.652) | 11.790(11.528-12.052) |
| **2000** | 6.703(6.830-6.823) | 3.508(3.397-3.620) | 11.753(11.493-12.013) |
| **2001** | 6.720(6.600-6.839) | 3.524(3.413-3.636) | 11.721(11.464-11.977) |
| **2002** | 6.859(6.739-6.978) | 3.712(3.598-3.826) | 11.742(11.488-11.996) |
| **2003** | 6.662(6.545-6.779) | 3.482(3.372-3.592) | 11.547(11.298-11.796) |
| **2004** | 6.850(6.732-6.968) | 3.517(3.408-3.626) | 11.901(11.600-12.151) |
| **2005** | 6.831(6.714-6.948) | 3.513(3.404-3.622) | 11.908(11.660-12.155) |
| **2006** | 6.792(6.677-6.907) | 3.453(3.346-3.560) | 11.885(11.641-12.129) |
| **2007** | 6.870(6.755-6.985) | 3.506(3.399-3.613) | 11.925(11.684-12.166) |
| **2008** | 6.815(6.702-6.928) | 3.486(3.380-3.591) | 11.766(11.529-12.002) |
| **2009** | 6.789(6.677-6.902) | 3.361(3.258-3.465) | 11.841(11.606-12.076) |
| **2010** | 6.898(6.786-7.011) | 3.431(3.327-3.535) | 11.999(11.766-12.233) |
| **2011** | 6.852(6.742-6.963) | 3.448(3.345-3.551) | 11.762(11.535-11.989) |
| **2012** | 6.774(6.665-6.882) | 3.306(3.206-3.405) | 11.768(11.544-11.993) |
| **2013** | 6.857(6.749-6.965) | 3.314(3.215-3.413) | 11.837(11.615-12.058) |
| **2014** | 6.704(6.598-6.810) | 3.252(3.155-3.349) | 11.528(11.312-11.744) |
| **2015** | 6.738(6.633-6.843) | 3.329(3.232-3.426) | 11.500(11.287-11.713) |
| **2016** | 6.745(6.641-6.849) | 3.326(3.229-3.423) | 11.587(11.375-11.798) |
| **2017** | 6.577(6.476-6.678) | 3.300(3.204-3.396) | 11.199(10.995-11.404) |
| **2018** | 6.431(6.332-6.530) | 3.139(3.048-3.231) | 10.954(10.755-11.153) |
| **2019** | 6.322(6.226-6.419) | 3.131(3.040-3.222) | 10.751(10.556-10.946) |
| **2020** | 6.159(6.065-6.254) | 2.961(2.874-3.047) | 10.532(10.342-10.723) |
| **Total** | 6.686(6.663-6.709) | 3.382(3.360-3.404) | 11.574(11.525-11.623) |

**Age-Adjusted Rate (95% CI)**

**Supplementary Table S5.Bladder cancer-associated AAMR race-stratified per 100,000 people in the United States 1999-2020.**

| **Year** | **Hispanic or Latino** | **Black or African American** | **White people** |
| --- | --- | --- | --- |
| **1999** | 2.803(2.298-3.309) | 6.161(5.757-6.564) | 6.906(6.776-7.036) |
| **2000** | 2.343(1.893-2.792) | 5.768(5.377-6.159) | 6.946(6.816-7.076) |
| **2001** | 3.152(2.654-3.649) | 5.732(5.348-6.117) | 6.941(6.812-7.070) |
| **2002** | 3.094(2.618-3.571) | 6.11(5.714-6.506) | 7.047(6.918-7.176) |
| **2003** | 2.616(2.194-3.038) | 5.806(5.423-6.188) | 6.888(6.762-7.015) |
| **2004** | 2.558(2.147-2.969) | 5.666(5.291-6.040) | 7.109(6.981-7.236) |
| **2005** | 2.964(2.531-3.396) | 5.796(5.422-6.170) | 7.097(6.970-7.224) |
| **2006** | 2.519(2.139-2.899) | 5.934(5.560-6.308) | 7.073(6.947-7.198) |
| **2007** | 2.500(2.130-2.869) | 5.900(5.532-6.268) | 7.151(7.026-7.276) |
| **2008** | 2.940(2.520-3.328) | 5.206(4.867-5.545) | 7.135(7.012-7.259) |
| **2009** | 2.753(2.386-3.120) | 5.519(5.173-5.866) | 7.085(6.962-7.208) |
| **2010** | 2.919(2.500-3.288) | 5.702(5.352-6.052) | 7.249(7.126-7.373) |
| **2011** | 2.582(2.249-2.916) | 5.533(5.199-5.868) | 7.195(7.073-7.317) |
| **2012** | 2.666(2.337-2.994) | 5.338(5.013-5.663) | 7.130(7.010-7.250) |
| **2013** | 2.766(2.444-3.089) | 5.408(5.088-5.727) | 7.190(7.071-7.309) |
| **2014** | 2.747(2.438-3.057) | 5.571(5.250-5.892) | 7.037(6.920-7.154) |
| **2015** | 2.645(2.350-2.940) | 5.185(4.882-5.488) | 7.132(7.016-7.249) |
| **2016** | 2.753(2.461-3.044) | 5.375(5.070-5.680) | 7.126(7.010-7.241) |
| **2017** | 2.633(2.355-2.911) | 5.232(4.937-5.527) | 6.991(6.877-7.104) |
| **2018** | 2.534(2.270-2.799) | 5.116(4.831-4.401) | 6.805(6.695-6.915) |
| **2019** | 2.666(2.404-2.928) | 4.929(4.651-5.206) | 6.712(6.604-6.820) |
| **2020** | 2.606(2.352-2.859) | 4.695(4.431-4.960) | 6.537(6.432-6.643) |
| **Total** | 2.688(2.615-2.760) | 5.482(5.411-5.553) | 7.003(6.977-7.028) |

**Age-Adjusted Rate (95% CI)**

**Supplementary Table S6.Bladder cancer-associated AAMR urban-rural classification-stratified per 100,000 people in the United States 1999-2020.**

| **Year** | **Metropolitan** | **Non-Metropolitan** |
| --- | --- | --- |
| **1999** | 6.818(6.683-6.954) | 6.473(6.200-6.746) |
| **2000** | 6.736(6.602-6.870) | 6.609(6.335-6.883) |
| **2001** | 6.793(6.660-6.926) | 6.485(6.215-6.756) |
| **2002** | 6.878(6.740-7.011) | 6.710(6.436-6.984) |
| **2003** | 6.643(6.140-6.772) | 6.653(6.383-6.924) |
| **2004** | 6.838(6.708-6.969) | 6.948(6.672-7.224) |
| **2005** | 6.865(6.736-6.995) | 6.741(6.471-7.012) |
| **2006** | 6.810(6.682-6.938) | 6.874(6.602-7.145) |
| **2007** | 6.875(6.748-7.003) | 6.921(6.650-7.191) |
| **2008** | 6.826(6.701-6.951) | 6.885(6.616-7.154) |
| **2009** | 6.710(6.587-6.830) | 7.021(6.752-7.290) |
| **2010** | 6.844(6.721-6.968) | 7.223(6.950-7.496) |
| **2011** | 6.785(6.664-6.906) | 7.122(6.854-7.389) |
| **2012** | 6.700(6.581-6.819) | 7.149(6.883-7.416) |
| **2013** | 6.802(6.683-6.920) | 7.058(6.794-7.321) |
| **2014** | 6.661(6.545-6.777) | 6.910(6.651-7.170) |
| **2015** | 6.632(6.518-6.747) | 7.265(7.002-7.528) |
| **2016** | 6.722(6.609-6.836) | 6.842(6.588-7.097) |
| **2017** | 6.454(6.344-6.564) | 7.219(6.960-7.478) |
| **2018** | 6.275(6.168-6.381) | 6.994(6.742-7.245) |
| **2019** | 6.219(6.114-6.324) | 6.849(6.601-7.097) |
| **2020** | 6.025(5.923-6.127) | 6.731(6.487-6.975) |
| **Total** | 6.640(6.614-6.665) | 6.887(6.831-6.944) |

**Age-Adjusted Rate (95% CI)**

**Supplementary Table S7.Bladder cancer-associated AAMR census region-stratified per 100,000 people in the United States 1999-2020.**

| **Census Region** | **Year** | **Age-Adjusted Rate with 95% CI** |
| --- | --- | --- |
| **Census Region 1: Northeast** | 1999 | 7.570(7.291-7.849) |
| **Census Region 1: Northeast** | 2000 | 7.541(7.264-7.817) |
| **Census Region 1: Northeast** | 2001 | 7.533(7.257-7.809) |
| **Census Region 1: Northeast** | 2002 | 7.477(7.204-7.750) |
| **Census Region 1: Northeast** | 2003 | 7.123(6.859-7.388) |
| **Census Region 1: Northeast** | 2004 | 7.446(7.176-7.717) |
| **Census Region 1: Northeast** | 2005 | 7.209(6.944-7.474) |
| **Census Region 1: Northeast** | 2006 | 7.410(7.142-7.677) |
| **Census Region 1: Northeast** | 2007 | 7.569(7.301-7.837) |
| **Census Region 1: Northeast** | 2008 | 7.454(7.190-7.718) |
| **Census Region 1: Northeast** | 2009 | 7.362(7.101-7.624) |
| **Census Region 1: Northeast** | 2010 | 7.647(7.381-7.913) |
| **Census Region 1: Northeast** | 2011 | 7.438(7.178-7.699) |
| **Census Region 1: Northeast** | 2012 | 7.304(7.049-7.560) |
| **Census Region 1: Northeast** | 2013 | 7.209(6.957-7.461) |
| **Census Region 1: Northeast** | 2014 | 7.269(7.018-7.520) |
| **Census Region 1: Northeast** | 2015 | 7.160(6.912-7.407) |
| **Census Region 1: Northeast** | 2016 | 7.184(6.937-7.431) |
| **Census Region 1: Northeast** | 2017 | 6.883(6.646-7.120) |
| **Census Region 1: Northeast** | 2018 | 6.619(6.388-6.850) |
| **Census Region 1: Northeast** | 2019 | 6.360(6.136-6.583) |
| **Census Region 1: Northeast** | 2020 | 6.056(5.839-6.273) |
| **Census Region 2: Midwest** | 1999 | 6.977(6.724-7.229) |
| **Census Region 2: Midwest** | 2000 | 6.787(6.540-7.035) |
| **Census Region 2: Midwest** | 2001 | 6.954(6.704-7.203) |
| **Census Region 2: Midwest** | 2002 | 6.877(6.631-7.124) |
| **Census Region 2: Midwest** | 2003 | 6.982(6.735-7.229) |
| **Census Region 2: Midwest** | 2004 | 7.105(6.857-7.353) |
| **Census Region 2: Midwest** | 2005 | 7.009(6.765-7.254) |
| **Census Region 2: Midwest** | 2006 | 7.026(6.782-7.270) |
| **Census Region 2: Midwest** | 2007 | 7.021(6.779-7.263) |
| **Census Region 2: Midwest** | 2008 | 7.248(7.004-7.492) |
| **Census Region 2: Midwest** | 2009 | 7.107(6.867-7.348) |
| **Census Region 2: Midwest** | 2010 | 7.182(6.942-7.422) |
| **Census Region 2: Midwest** | 2011 | 7.082(6.846-7.319) |
| **Census Region 2: Midwest** | 2012 | 6.911(6.679-7.143) |
| **Census Region 2: Midwest** | 2013 | 7.260(7.025-7.496) |
| **Census Region 2: Midwest** | 2014 | 6.912(6.684-7.141) |
| **Census Region 2: Midwest** | 2015 | 7.091(6.861-7.321) |
| **Census Region 2: Midwest** | 2016 | 6.994(6.767-7.221) |
| **Census Region 2: Midwest** | 2017 | 6.798(6.578-7.018) |
| **Census Region 2: Midwest** | 2018 | 6.697(6.479-6.914) |
| **Census Region 2: Midwest** | 2019 | 6.612(6.399-6.824) |
| **Census Region 2: Midwest** | 2020 | 6.431(6.222-6.639) |
| **Census Region 3: South** | 1999 | 6.196(6.000-6.392) |
| **Census Region 3: South** | 2000 | 6.456(6.257-6.655) |
| **Census Region 3: South** | 2001 | 6.226(6.032-6.420) |
| **Census Region 3: South** | 2002 | 6.645(6.446-6.844) |
| **Census Region 3: South** | 2003 | 6.260(6.069-6.451) |
| **Census Region 3: South** | 2004 | 6.473(6.280-6.666) |
| **Census Region 3: South** | 2005 | 6.575(6.382-6.767) |
| **Census Region 3: South** | 2006 | 6.583(6.393-6.773) |
| **Census Region 3: South** | 2007 | 6.598(6.409-6.786) |
| **Census Region 3: South** | 2008 | 6.520(6.335-6.706) |
| **Census Region 3: South** | 2009 | 6.580(6.396-6.764) |
| **Census Region 3: South** | 2010 | 6.613(6.430-6.796) |
| **Census Region 3: South** | 2011 | 6.534(6.354-6.713) |
| **Census Region 3: South** | 2012 | 6.573(6.396-6.751) |
| **Census Region 3: South** | 2013 | 6.637(6.461-6.813) |
| **Census Region 3: South** | 2014 | 6.585(6.412-6.758) |
| **Census Region 3: South** | 2015 | 6.562(6.391-6.733) |
| **Census Region 3: South** | 2016 | 6.636(6.467-6.805) |
| **Census Region 3: South** | 2017 | 6.479(6.314-6.644) |
| **Census Region 3: South** | 2018 | 6.333(6.173-6.492) |
| **Census Region 3: South** | 2019 | 6.283(6.126-6.440) |
| **Census Region 3: South** | 2020 | 6.237(6.082-6.392) |
| **Census Region 4: West** | 1999 | 6.497(6.229-6.766) |
| **Census Region 4: West** | 2000 | 6.170(5.911-6.428) |
| **Census Region 4: West** | 2001 | 6.577(6.313-6.842) |
| **Census Region 4: West** | 2002 | 6.541(6.281-6.801) |
| **Census Region 4: West** | 2003 | 6.467(6.212-6.723) |
| **Census Region 4: West** | 2004 | 6.648(6.391-6.904) |
| **Census Region 4: West** | 2005 | 6.751(6.495-7.006) |
| **Census Region 4: West** | 2006 | 6.457(6.209-6.704) |
| **Census Region 4: West** | 2007 | 6.551(6.305-6.797) |
| **Census Region 4: West** | 2008 | 6.301(6.063-6.540) |
| **Census Region 4: West** | 2009 | 6.186(5.953-6.419) |
| **Census Region 4: West** | 2010 | 6.455(6.218-6.691) |
| **Census Region 4: West** | 2011 | 6.57(6.336-6.804) |
| **Census Region 4: West** | 2012 | 6.484(6.254-6.713) |
| **Census Region 4: West** | 2013 | 6.507(6.279-6.734) |
| **Census Region 4: West** | 2014 | 6.153(5.936-6.371) |
| **Census Region 4: West** | 2015 | 6.304(6.088-6.520) |
| **Census Region 4: West** | 2016 | 6.316(6.102-6.529) |
| **Census Region 4: West** | 2017 | 6.233(6.023-6.43) |
| **Census Region 4: West** | 2018 | 6.037(5.834-6.239) |
| **Census Region 4: West** | 2019 | 6.011(5.811-6.21) |
| **Census Region 4: West** | 2020 | 5.854(5.659-6.049) |

**Supplementary Table S8.Bladder cancer-associated AAMR state-stratified per 100,000 people in the United States 1999-2020.**

| **State** | **Age-Adjusted Rate with 95% CI** |
| --- | --- |
| **Maine** | 8.868(8.495-9.242) |
| **Vermont** | 8.229(7.682-8.776) |
| **Nevada** | 7.983(7.683-8.282) |
| **Delaware** | 7.937(7.479-8.394) |
| **New Hampshire** | 7.835(7.457-8.213) |
| **Rhode Island** | 7.803(7.403-8.204) |
| **Ohio** | 7.757(7.632-7.882) |
| **Massachusetts** | 7.558(7.395-7.720) |
| **West Virginia** | 7.380(7.089-7.672) |
| **New Jersey** | 7.368(7.227-7.509) |
| **Kentucky** | 7.336(7.129-7.544) |
| **Pennsylvania** | 7.303(7.194-7.413) |
| **Oregon** | 7.295(7.083-7.507) |
| **Michigan** | 7.243(7.111-7.375) |
| **Indiana** | 7.199(7.031-7.367) |
| **Connecticut** | 7.167(6.954-7.379) |
| **Maryland** | 7.127(6.945-7.308) |
| **Washington** | 7.073(6.906-7.240) |
| **Montana** | 7.052(6.653-7.451) |
| **Wisconsin** | 7.005(6.835-7.175) |
| **Oklahoma** | 6.891(6.677-7.106) |
| **Idaho** | 6.855(6.510-7.200) |
| **Florida** | 6.763(6.680-6.846) |
| **Illinois** | 6.759(6.643-6.875) |
| **Total** | 6.686(6.663-6.709) |
| **District of Columbia** | 6.659(6.107-7.211) |
| **New York** | 6.653(6.562-6.743) |
| **Virginia** | 6.602(6.452-6.752) |
| **Tennessee** | 6.600(6.437-6.763) |
| **Arkansas** | 6.586(6.353-6.818) |
| **Georgia** | 6.562(6.415-6.709) |
| **Iowa** | 6.520(6.305-6.735) |
| **Missouri** | 6.465(6.305-6.625) |
| **North Carolina** | 6.388(6.255-6.521) |
| **Arizona** | 6.344(6.188-6.501) |
| **Wyoming** | 6.338(5.787-6.889) |
| **North Dakota** | 6.331(5.875-6.786) |
| **South Carolina** | 6.271(6.085-6.457) |
| **Louisiana** | 6.219(6.027-6.411) |
| **Minnesota** | 6.210(6.040-6.380) |
| **Kansas** | 6.198(5.969-6.428) |
| **California** | 6.141(6.072-6.209) |
| **Alabama** | 6.130(5.952-6.309) |
| **Nebraska** | 6.076(5.795-6.370) |
| **South Dakota** | 6.012(5.604-6.357) |
| **Colorado** | 5.963(5.774-6.152) |
| **Alaska** | 5.944(5.306-6.582) |
| **New Mexico** | 5.833(5.561-6.106) |
| **Texas** | 5.739(5.653-5.825) |
| **Mississippi** | 5.676(5.450-5.901) |
| **Utah** | 5.428(5.158-5.699) |
| **Hawaii** | 4.292(4.025-4.560) |
